# Medical student stress: an elective course as a possibility of help

**DOI:** 10.1186/s13104-015-1399-y

**Published:** 2015-09-10

**Authors:** Maria Amélia Dias Pereira, Maria Alves Barbosa, Jomar Cleison de Rezende, Rodolfo Furlan Damiano

**Affiliations:** Faculdade de Medicina da Universidade Federal de Goiás, Primeira Avenida, s/n, Setor Leste Universitário, Goiânia, Goiás CEP: 74605-020 Brazil; Faculdade de Enfermagem da Universidade Federal de Goiás, Rua 227, quadra 68, s/n, Setor Leste Universitário, Goiânia, Goiás CEP: 74605-080 Brazil; Hospital das Clínicas da Universidade Federal de Goiás, Primeira Avenida, s/n, Setor Leste Universitário, Goiânia, Goiás CEP: 74605-020 Brazil; Faculdade de Ciências Médicas e da Saúde da Pontifícia Universidade Católica de São Paulo (FCMS-PUCSP), Rua Joubert Wey, 290, Sorocaba, São Paulo CEP: 18030-070 Brazil

**Keywords:** Professional stress, Coping strategies, Medical students, Elective course

## Abstract

**Background:**

The frequently observed stress of medical students worldwide leads them to have psychic suffering often leading to illness. Minor psychic disorders such as anxiety, depression and burnout, have a higher prevalence in these students than in the rest of random population. Different initiatives were tried to minimize the deleterious effects of the medical course and this article aims at showing the repercussions of a elective course that and was proposed as a possibility to help the students.

**Methods:**

A qualitative case study took place in a public Brazilian university as an elective discipline offered to medical students in 2013, offering coping strategies for professional stress. The data was collected through a semi-structured individual questionnaire that was anonymous, and given to students on the last day of the course, with 18 Likert scale questions about personal and behavioural changes observed after taking the course. Objective questions were asked about their perception of stress at the beginning and at the end of the course: the use of the coping strategies taught and the perception of the utility of the content. In addition, one open-ended question was asked about the meaning of the discipline to the students. The quantitative data was analysed with descriptive simple statistics and the qualitative with the support of the WebQDA software. The research project was approved by the ethics committee of the institution.

**Results:**

The results showed that the course contributed positively to the students’ academic life: 67 % reported less symptoms of stress at the end of the course; 76 % adopted new coping strategies; and 90 % considered that this learning activity was useful for identifying stressors and sharing them with colleagues.

**Conclusions:**

The elective course produced benefits to the students, representing theoretical-practical learning and an opportunity for reflection and self-knowledge, which caused psychological, behavioural and lifestyle changes. It is recommended that further studies on this theme should be conducted.

## Background

Medical students’ mental health has been exhaustively studied [1–3]. Stress is one of the most important issues, which have been reported with high prevalence, sometimes higher than in the general population [4]. Studies on Burnout [5], depression [6, 7] and anxiety [8] also reported a worrying prevalence. Minor health disorders were evaluated in several medical schools, showing a significant prevalence [9, 10].

The literature shows that stress symptoms are extremely frequent among medical students. In a Brazilian study it was found that 65.7 % of the students in a Brazilian medical school had symptoms of stress, mainly during the residency phase, and that a greater repertoire of social skills (which includes assertiveness and empathy) is related to a lower rate of stress in men than in women, although this difference was not significant [11]. In this study, the main stressors cited by the students were the following (in descending order): unfair professors; excessive numbers of subjects to study; a huge number of examinations; oral examinations; lack of leisure time; expectations on the medical profession; and fear of failing in their studies.

Trindade e Vieira [12] studied stressful situations and the students’ defence mechanisms in difficult and embarrassing situations regarding patients, showing the importance of such knowledge to favour the creation of measures to better guide the students in their daily life and to avoid abuse by faculty members, patients and family.

In a study with medical students of a Brazilian public university [13], the stress coping strategies developed by the students themselves included valuation of interpersonal relationships and daily life phenomena, balance between studies and leisure, time organisation, attention to health, diet and sleep, physical activities, religiosity, tailoring the personality to deal with adverse situations, and seeking psychological assistance.

A few studies investigated interventions for students to minimise the distress inherent to medical courses [14–16]. In a systematic review of stress management in medical education [17], more than 600 studies were selected, and only 24 reported intervention and support programmes for medical students and/or residents. The authors concluded that these programmes have brought benefits such as improvement in the students’ immune systems, decrease in depression and anxiety, increase in spirituality and empathy, better knowledge of alternative therapies for future use, better knowledge of stress and better use of positive coping skills, thereby reinforcing the importance and need of support services for students.

Data found in the literature [18] and the faculty’s perception of the psychological distress of medical students that we found in our work environment [19] have motivated us to provide some form of help and prevention regarding mental health. In 2011, we elaborated an elective course that was created and delivered for medical students in a Brazilian midwest public university. The results of this research were published in 2013 [20], indicating that it was a valid form of help and support to the students enrolled on it. The elective course consisted of a series of content aimed at allowing the students to freely widen their academic backgrounds [21].

In 2013, we decided to reply and change the format of this elective course. We chose a mixed approach in which some classes were aimed at teaching techniques based on cognitive-behavioural theories, such as Jacobson’s progressive relaxation [22], diaphragmatic breathing, cognitive restructuring, and social skills training (e.g. assertiveness). The other classes were based on psychoanalytic references, such as reading articles by psychoanalysts [23–27], and addressed more reflective themes such as physician identity, medical vocation, ego defence mechanisms, work psychodynamics, and social networks. It is thought that when one understands what happens with an individual, it makes it easier to cope with stressful situations, and naming what causes suffering allows the individual to elaborate and create changing conditions.

The present article aimed to identify personal changes that occurred in the students who attended the elective course ‘Strategies of Coping with Professional Stress’, to analyse their perception of the stress symptoms before and after the course, to assess their use of coping strategies learned, and to evaluate their perceptions of the use of content and the meaning of the course.

## Methods

Qualitative study aimed to investigate the repercussion of an elective course developed as an attempt to reduce medical students' psychological distress.

In 2011 it was approved a research project (protocol number 027/2011) which aimed at offering to medical students of a midwest public Brazilian university an elective course named “Strategies of Coping with Professional Stress”, and later evaluate students` opinion regarding the intervention and the changes in the their life after the course. In 2012 it was sent to the Ethics Committee an addendum to the initial project, that was later approved, to repeat the course in 2013, reapplying the same questionnaire of evaluation of the course and its repercussions to the students enrolled in this second version. In this paper we will address only part of the questionnaire applied in 2013 course.

The sample consisted of 76 students who were second-, third- and fourth-year undergraduate medical students. The inclusion criteria were being enrolled in the elective course and completing it.

Data collection was performed by means of a semi-structured questionnaire (attached) containing 18 Likert-type questions on personal changes, 6 closed questions (with justifications) on stress symptoms before and after the course, the use of coping strategies learned during the course and perceptions of the content, interest and participation in the course, expectations, and an open-ended question on the meaning of the elective course for the students. The questionnaire was developed by the authors solely for the application in this research and was given to the students in the last day of class. Only students who agreed with the consent form were abled to fill out the questionnaire.

The students also were given the Lipp stress symptom inventory [28] at the beginning of the course to investigate their symptoms, thus allowing us to evaluate whether they had stress. The students responded to the questionnaire individually and anonymously. The questionnaires, however, were not considered by professors/researchers because they served only to sensitise the students to their possible symptoms and conditions. After 4 months, the same questionnaire was applied again so that the students could evaluate whether their stress symptoms had changed by the end of the course.

The themes were addressed by two classrooms of approximately 40 students each, by using an active learning methodology, and attendance was fortnightly. The content was as follows: the concept of both stress and coping strategies; vicissitudes of the medical course; medical-course-related stressors; medical students’ psychological distress; the concept of quality of life; stress coping strategies used by medical students; cognitive restructuring; Jacobson’s progressive relaxation (practice); medical vocation; personality/resilience; ego defence mechanisms; work psychodynamics; assertiveness and communication; and social networks.

Quantitative data were presented and analysed by using single descriptive statistics, whereas qualitative data were organised and grouped into previously established categories. Two dimensions emerged from these categories, namely, the learning and impact of the course. Analysis of the content followed the methodology suggested by Bardin [29], with fluctuating reading and then selection of ideas, grouping into categories and dimensions, statistical operations, synthesis and selection of results, and inferences and interpretations.

## Results

Among the 76 medical students who participated and completed the elective course in 2013, 40 (52.63 %) were women and 36 (47.37 %) were men, with a mean age of 21. Of these, 60 (78.94 %) were second-year undergraduates, 12 (15.78 %) were third-year undergraduates and 4 (5.28 %) were fourth-year undergraduates.

### Students’ perceptions on their own behavioural changes

**Fig. 1:**
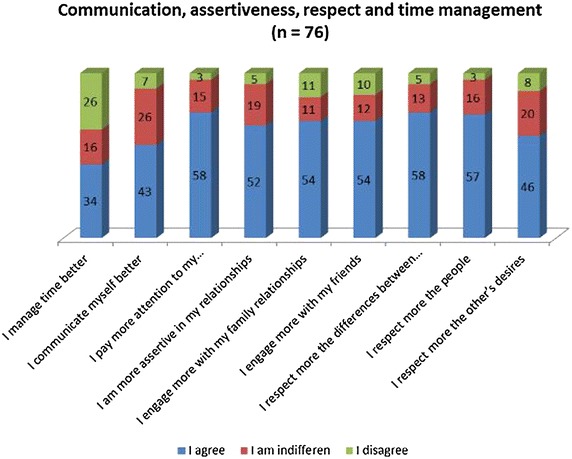
Communication, assertiveness, respect and time management (n = 76)

**Fig. 2:**
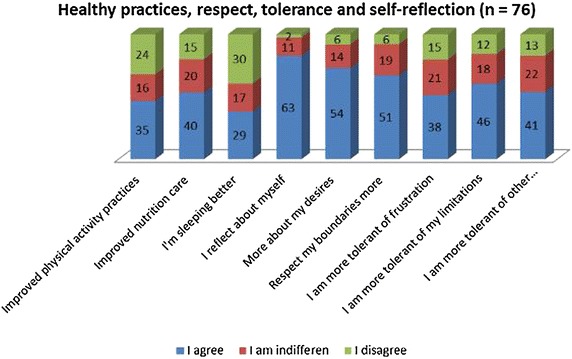
Students’ perception on time management, communication, assertiveness, relationships and respect

Likert-type questions were presented as affirmative statements for which the students had to answer whether they agreed or disagreed (fully or partially) or if they were indifferent, with the changes expected following the intervention by means of the elective course. The results are listed in Figs. 1 and 2; n = 76.

The students reported that they started to observe more about themselves and to reflect and respect their desires and limitations. The majority of them agreed that they are more assertive in their relationships, more engaged with interactions with family and friends, pay more attention to their own feelings, and started respecting people and personal differences more. However, sleeping better was an issue not agreed on by the majority.

Our study showed that almost half the students stated that they improved their taking part in physical activities during the semester and they recognised that they could manage time better. Becoming more tolerant to frustrations was also reported by 50 % of the students.

With regard to other items, as seen in Figs. 1 and 2, there was agreement by the great majority of the students. Some items were very well rated by the students, such as self-reflection (86 %), respect to differences (76 %), people (75 %), and own desires (71 %). The percentage of disagreeing students was small, and considering that all statements showed favourable changes, one can conclude that the intervention was beneficial to the students.

### Stress symptoms perceived before and after the intervention

Out of a total of 76 students, 51 (67.1 %) responded that their stress symptoms decreased by the end of the course (Table 1).

**Table 1:** Symptoms of stress before and after the course

Perception of the symptoms	Men	Women	Total N/%
More stress before the course	24	27	51/67.1 %
No difference	9	0	9/11.8 %
More stress at the end of the course	6	10	16/21.1 %
Total	39	37	76/100 %

Among the explanations given by the students for these responses, most refer to the coping strategies learned during the course.

‘The identification of the symptoms and the classroom dynamics enabled me to improve my state of stress from the first moment.’ (S.4 – S = Student)

‘Although the causes of stress have not changed, I’ve started reflecting better on them. This happened because my participation in this course made me turn my attention to stressful factors that were not noticed before.’ (S.38)

Some students perceived a reduction in their stress symptoms after having made changes in their lifestyle that had positive results, including seeking professional help.

‘I consider that over the semester I learned how to better manage my time, and I’m less stressed.’ (S.44)

‘Greater acquaintance with colleagues, therapeutic help, and leisure hours helps me decrease stress.’ (S.36)

Other students (25.5 %) recognised that external changes really provided moments of low stress, such as the end of a semester or improved courses.

‘Because it’s the end of semester, we are already more relaxed as we passed the majority of the subjects, and vacations are getting closer. All these are relaxing factors.’ (S.45)

In the present study, 16 students responded that they had more symptoms of stress at the end of the course, with 10 being female. Most (70.5 %) reported impairment due to the end-of-year pressures, whereas others even recognised the benefits despite their responses.

‘The medical course became even heavier with difficult subjects, higher number of evaluations, overload of studies, and higher amount of work.’ (S.7)

‘When the medical course began, the academic activities were easy as there were fewer examinations or work, differently from the end of the semester. However, I can surely say that I’m much better than the past years during the same period.’ (S.11)

Among the 76 respondents, nine (11.8 %) students—all male—reported that they perceived no difference in their stress symptoms before or after the intervention, either because they were always stressed or because they did not follow the strategies learned or never felt stressed.

‘I’ve perceived no difference because I’m still stressed.’ (S.26)

‘Nothing’s changed, since I had few problems.’ (S.71)

With regard to the symptoms of stress, the students’ perceptions of those who completed the course suggested contributions from this intervention, although the problems still remained.

### Use of stress coping strategies by students after the course

In the present study, the majority of the students (76.3 %) reported they had incorporated new coping strategies (Table 2).

**Table 2:** Use of strategies of coping with stress

Use of Strategies	Men	Women	Total N/%
I’ve incorporated new coping strategies	27	31	58/76.3 %
Everything I do today I have done before	6	6	12/15.8 %
I still do not use any strategy	6	0	6/7,9 %
Total	39	37	76/100 %

According to their statements, we can observe that some respondents changed their daily lifestyle, including more leisure time, physical activities, and relaxation techniques. For example:

‘I’m doing more physical activities, listening to more music, going to church, going out more often with friends.’ (S.1)

‘I use relaxation techniques for sleeping.’ (S.49)

During the course, a self-reflective activity was suggested where the students would list daily activities they do or don’t do and what they enjoy, in order to assess the possibility of changes. It was observed that most students reported they had made changes in this respect.

‘Realising that stress is a common thing encouraged me to ignore some actions that would previously be disturbing to me.’ (S.43)

‘I’ve learned to better manage my time.’ (S.53)

Other statements address personal changes, which may be considered as signs of maturity as well as the result of an improved self-knowledge.

‘I’m learning to be more assertive and value more my opinion.’ (S.17)

‘Despite thinking I still need to improve a lot, in some moments of stress I remember the course and try to put what I learned into action.’ (S.24)

Twelve students (15.7 %) reported that all of the strategies they have been currently using to minimise their stress had already been used by them before the course, recognising, however, the need to improve in some aspects.

‘Perhaps because I’ve been reading much about stress coping and having great family support, I’ve already used the strategies learned despite being extremely valued.’ (S.39)

‘I used to listen to music or sometimes I was not so rigid about my agenda so that I could alleviate my stress. Today, I go on doing the same thing. I did not find another strategy that could guide me.’ (S.75)

Six male students who reported they do not use any strategies stated that they had still not found anything which they could adapt; they had a lack of time or they did not believe it was possible to change.

‘Stress has been following me since the high school days because I always demanded myself to have good grades and good performance, thus becoming very perfectionist. This seems to be impossible to change as it seems intrinsic to my existence, being as unchangeable as the colour of my eyes.’ (S.7)

### Students’ perceptions of the course

The majority of the participants (90 %) considered that the course was useful, with 43 % students stating that it reinforced concepts and improved their stress coping practices, 20 considering that despite the usefulness of the learning they recognised they had not yet applied what they learned, and 14 reporting that they learned new strategies and are using them. Six students chose two items regarding this question. The results are listed in Table 3.

**Table 3:** Students’ perceptions of the course ‘Strategies of Coping with Professional Stress’

Perceptions of the course	Men	Women	Total N/%
There was no nothing new	2	0	2/2.4 %
Useful course as concepts were reinforced	19	24	43/52.4 %
Useful course, but I did not use it	13	7	20/24.3 %
Useful course as I have learned new strategies	9	5	14/17,0 %
My perception of stress improved/worsened	2	1	3/3.6 %
Total	45	37	82/100 %

Nevertheless, two students reported that there was nothing new and three considered that they became more stressed because of their improved perception of the stressors in undergraduate medical education.

### The meaning of the course to the participants

The answers given by the respondents regarding the meaning of the elective course were analysed by using the WebQDA software, whereas the ideas raised (number of times in parentheses) were grouped into six categories and then into two dimensions: learning and impact of the course. The results are listed in Table 4.

**Table 4:** Ideas, categories and dimensions of the statements made by the students regarding the meaning of the course

Ideas	Categories	Dimension
To understand new/interesting themes (7)	Theoretical learning	Learning
To have theoretical knowledge (4)		
To increase knowledge on mental health (2)		
To raise ideas regarding mental health (1)		
To learn stress coping strategies (22)	Practical learning	
To know about stress/course (16)		
To decrease/alleviate the stress (9)		
To be able to identify stressors (7)		
To have self-reflection/self-knowledge (21)	Moment of reflection	Impacts of the course
To share feelings with colleagues (12)	Benefits of the course	
To have time for distraction/resting (7)		
To perceive common feelings (5)		
To have moments of support/relief (3)		
To understand one’s limitations (3)	Psychological/emotional changes	
To become less anxious/stressed (3)		
To decrease the demands (1)		
To improve relationships (1)		
To be aware of what was already known (1)		
To improve the quality of life (8)	Lifestyle changes	
To better take care of one’s self (6)		
o change lifestyle (3)		
To improve performance (1)		

The participants gave more far-reaching answers: some were more succinct in their statements, and others answered in more detail, with only one student leaving the question blank.

Many students reinforced the idea that the course was an important event, being useful, pleasant or very good; some reported that the course overcame their expectations; and others suggested the course should be part of the mandatory curriculum. Because it is an elective course, which is exclusively offered to medical students and aimed at assisting them with regard to their well-being, we actually expected that the course would be highly evaluated by the students, but it is important that they have room to express their opinions without needing to identify themselves.

‘As a second-year undergraduate student, considered the worst of the six undergraduate years, I can surely say that this was the best elective course I’ve attended this year. In my opinion, it should be part of the mandatory curriculum.’ (S.8)

‘This course was of extreme importance to me as I did it at a moment of much stress and I managed to apply it and reflect on my stance as student, son and friend.’ (S.54)

Even for those students who completed the course stating that it was only an opportunity for them to fulfil their schedules, one could observe that there were some extra benefits as they commented on their learning, reflections, or moments of integration. At the beginning of the course, when the students were asked about the reason why they decided to participate, it became clear that the great majority of the participants were doing it because of a curriculum necessity. If they had not been obliged to fill their free schedules, it is likely that we would not have had such a high number of participants. It is worth emphasising that 50 places were initially offered, but 100 people appeared on the first day of enrolment for the course. Was it a demonstration of interest in the theme? Or did they already expect a less demanding course?

Some students recognised that the course had interesting strategies and they regretted not dedicating themselves enough to the practices proposed.

‘The course guided me to the way I should go, but I believe much of what was learned during the classes still needs to be put into practice.’ (S.27)

#### Dimension 1: learning

Most students reported that the meaning of the course was both theoretical and practical.

‘The course complemented my knowledge on the mental health of medical students.’ (S.5)

One can notice that many medical students have difficulty in recognising their fragility, and therefore they address all the issues impersonally and the themes theoretically, as if it was something about which they had no personal concern. Probably, it is a defence mechanism to avoid contact with the distress inherent to their human and limited condition.

‘It’s just more knowledge. Just learning.’ (S.60)

Themes motivating discussions and reflections among the students were included in the present study, and the great majority of them reported some benefits resulting from reflections and behavioural changes.

‘It enabled me to identify the stressors in the daily life, thus allowing me to cope with them by means of various practices learned.’ (S.6)

Some coping strategies learned were experienced in the classroom, such as Jacobson’s progressive muscle relaxation technique [22] and diaphragmatic breathing, which were analysed by two cognitive-behavioural psychologists.

It was observed that some students could benefit from these strategies:

‘I thought it was very good. I’ve learned relaxation techniques and felt more motivated to take care of myself.’ (S.57)

‘The course helped me see that some simple actions, such as breathing better, can decrease the stress and make me sleep better.’ (S.3)

Different benefits were observed as psychoanalysis allows self-knowledge and self-reflection that can change the way of being and seeing the world, whereas the cognitive-behavioural changes provide the individual with resources to cope with daily life situations more adequately.

‘It meant a serious reflection on the reasons for my stress and on how to deal with that in order to improve my quality of life and health.’ (S.56)

‘Now I can identify mistakes I make when I try to deal with stress and I learned better ways of facing daily life facts that can be stressful.’ (S.76)

### Dimension 2: impact of the course

By proposing a course where the students are given time and space to express their complaints, we expected that the participants could immediately benefit. Sharing common feelings and difficulties experienced in the undergraduate medical course with colleagues can, per se, bring relief and well-being to the students, and the results found confirm this expectation.

‘It’s a manner to discuss with other students about my impression on the world and theirs too.’ (S.30)

‘The aim was to seek knowledge and share experiences to allow me to cope with life stress factors more confidently.’ (S.43)

‘It’s an opportunity to learn ways of self-knowledge and to seek improvements that sometimes are not possible and then left aside.’ (S.22)

‘Besides being able to see that many suffer the same distress as me, it was good to know that there are strategies to cope with that.’ (S.24)

‘It’s a new space in the undergraduate medical education which is extremely necessary for us to find support for our major complaints.’ (S.9)

As a consequence of the course, the students report that they are now considering themselves more, and paying more attention to their feelings and emotions. Because of this attitude, other changes were perceived and reported, such as being less self-demanding, understanding and accepting their own limitations, and reducing anxiety.

‘It’s really a new way of facing my daily life situations, understanding my limitations, comprehending other persons, being more resolute and less melancholic.’ (S.35)

The aim in selecting these themes was to sensitise the students to daily life situations which normally are not addressed, although these are certainly felt by the majority of the medical students. As a result of self-awareness, some changes in lifestyle are made or at least planned. Awareness of habits harmful to health and situational abuse and disrespect leads to reflections on different choices. Many students who attended the course reported positive outcomes resulting from this differentiated view they gained.

‘It meant an initial kick-off for changes in my lifestyle, for thanks to this course I started thinking more about me and I sought a psychologist. I also started doing physical activities three times a week without remorse and being more assertive.’ (S.20)

## Discussion

These findings are corroborated by data from the literature.

A study by Dyrbye, Thomas and Shanafelt [5] found that teaching students stress coping strategies such as acceptance, planning, positive reappraisal, and self-distraction, can reduce psychological morbidity. The current research did not find morbidity, but the students reported changes in wellness and increases in quality of life.

The present study identified benefits similar to the ones found in a systematic review about stress management in medical education [17], such as the fact of acquiring better knowledge of the stress inherent to the medicine course and the incorporation of new stress coping strategies.

One can observe that for the great majority of the students who participated in the elective course, for different reasons, there was a reduction in their stress symptoms.

A similar study with nursing students demonstrated the efficacy of a training programme aimed at preventing stress [15].

In 2013, a meta-analysis of studies addressing mental health interventions for medical students was published [30]. The author selected 13 studies that had control groups and proper evaluation criteria and methods, concluding that interventions had a moderately positive effect on the students’ mental health compared to the non-intervention group. Also, short- and mid-term interventions were shown to be more favourable than long-term interventions (longer than four weeks). It is suggested to repeat the current research but with a shorter duration. However, in our study, we also observed a positive outcome with a longer intervention (4 months).

A study on the most used coping strategies by medical students of a public Brazilian university showed that the main ones were: positive reappraisal, self-control and social support. It was concluded that there exists the need to include the theme on stress in several undergraduate phases and obtain more humanistic behaviour models aimed at flexible coping [31]. We can consider that the course ‘Strategies of Coping with Professional Stress’ includes the suggestion of those authors.

It would be interesting to evaluate whether the students’ perceptions of the course had been maintained over some time, that is, if the benefits remained the same. A similar experience occurred in a Korean university, where the medical students were offered an elective course and 2 years later their perception was assessed [32]. Among the effects found elsewhere, the students reported that sharing their feelings with colleagues was a useful stress coping strategy and that the course enabled them to engage in pleasant activities without guilt. In our study it was also reported by some students that they reduced their feeling of guilt at the moment they started to prioritise other activities apart from the academic courses.

The Jacobson method of progressive relaxation is proven to be effective and the ideal would be to repeat this method during the course, but the students at least became aware of an available easy-to-use technique whose efficiency is shown in the literature [33]. The final evaluation of the course, done by the students enrolled on it, showed that there were real benefits with this technique.

An Iranian study [15] demonstrated the efficacy of a coping training programme in the reduction of symptoms of depression, anxiety and stress among nursing students. The programme consisted of eight sessions of 2 h each twice a week, and an evaluation instrument (Depression, Anxiety and Stress Scale—DASS-42) was used before and after the training. The results were compared to a control group, where significant differences were found following intervention. The strategies learned were information on stress, familiarity with progressive muscle relaxation and introduction to mental imagery, familiarity with the physical consequences of stress, diaphragmatic breathing training, observation of thoughts and feelings related to cognitive errors, and cognitive restructuring.

As in that study, our elective course also addressed the most common cognitive errors and taught the steps for cognitive restructuring. These techniques are tools that can be learned and become effective for some individuals. There were no tests in the current study to identify anxiety and depression before and after the intervention, but we can infer that the effects of the course would resemble the ones identified in the Iranian study.

Colares [34] conducted a social-drama research with medical students and found that sharing difficulties inherent to undergraduate medical education with colleagues was beneficial, favouring positive links among the participants as well as the experience of being in the other’s shoes by means of role-playing. All these enabled the students to have a more critical reflection on the development of their professional role. The students from the present research also stressed the importance of the opportunity to reflect provided in the course.

Researches have shown that allowing students to express themselves, reflect, and share their feelings increases their insights and decreases the probability of burnout [5]. In the present study, 82.8 % of the students stated to reflect more about themselves because of the course, which is expected to encourage healthier choices in life and less illness.

According to Colares [16], the reflective practice within contexts related to undergraduate medical education is an important tool for development of the students’ professional role, thus contributing to closing the gaps in the learning scenario. The students need strategies that can help them ‘process’ their academic experiences constructively and ethically.

De Marco et al. [14] worked with groups of reflection with fifth-year undergraduate students whose objective was prophylactic, that is, to create an activity that could aid in the detection, awareness and elaboration of the conflicts faced during the course of medicine, very similarly to that of our experience.

## Conclusion

Our elective course has contributed positively to the students’ life. The approach of the themes was shown to be useful as there was a reduction in their symptoms of stress and they also adopted new coping strategies. The course meant theoretical and practical learning for the students, thus allowing them moments of reflection and self-knowledge which led to psychological and emotional changes or even to changes in their lifestyle.

A space in the teaching institution where the students can express their subjectivity and interact with colleagues allows both pedagogical and prophylactic actions to alleviate the distress inherent in the process of undergraduate medical education.

The present study can be useful for professors, students and medical schools to implement interventions aimed to protect and minimise the harmful effects of undergraduate medical education. Creating opportunities for self-reflection and sharing feelings is one of the best ways of intervening with the psychological distress caused by academic situations.

One of the limitations of our study resides in the fact that the students’ opinions were expressed in the final questionnaire anonymously, but it was applied by the researchers who had themselves conducted the course.

Another limitation of our elective course, whose aim was to minimise psychological distresses identified during the undergraduate medical education, is that the ideal would be to have the themes discussed throughout the undergraduate medical course. Moreover, this format with space and moments for sharing and reflections among the students should also be available over the medical curriculum rather than in only one semester, as it was seen here.

We recommend that after 2 years the students who participated in the present study should be re-evaluated on their symptoms of stress and coping strategies.

## References

[1] Cerchiari EAN, Caetano D, Faccenda O (2005). Prevalência de transtornos mentais menores em estudantes universitários. Estudos de Psicologia..

[2] Facundes VLD, Ludermir AB (2005). Common mental disorders among health care students. Rev Bras de Psiquiatria..

[3] Benevides-Pereira AMT, Gonçalves MB (2009). Transtornos emocionais e a formação em medicina: um estudo longitudinal. Rev Bras Educ Med..

[4] Firth J (1986). Levels and sources of stress in medical students. Br Med J.

[5] Dyrbye LN, Thomas MR, Shanafelt TD (2006). Systematic review of depression, anxiety, and other indicators of psychological distress among US and Canadian medical students. Acad Med.

[6] Baldassin S (2010). Ansiedade e depressão no estudante de medicina: revisão de estudos brasileiros. Cadernos ABEM..

[7] Oliva-Costa EF, Santana YS, Santos ATRDA, Martins LAN, Melo EVD, Andrade TMD (2012). Sintomas depressivos entre internos de medicina em uma universidade pública brasileira. Revista da Associação Médica Brasileira.

[8] Baldassin S, Martins LC (2006). Andrade AGd. Traços de ansiedade entre estudantes de medicina. Arq Méd ABC..

[9] Lima MCP, Domingues MS, Cerqueira ATAR (2006). Prevalence and risk factors of common mental disorders among medical students. Rev Saúde Pública..

[10] Rocha ES, Sassi AP (2013). Transtornos mentais menores entre estudantes de medicina. Rev Bras Educ Med..

[11] Furtado ES, Falcone EMO, Clark C (2003). Avaliação do estresse e das habilidades sociais na experiência acadêmica de estudantes de medicina de uma universidade do Rio de Janeiro. Interação em Psicologia.

[12] Trindade LMDF, Vieira MJ (2013). O aluno de medicina e estratégias de enfrentamento no atendimento ao paciente. Rev Bras Educ Med..

[13] Zonta R, Robles ACC, Grosseman S (2006). Stress coping strategies developed by medical students of the Federal University of Santa Catarina. Rev Bras Educ Med..

[14] De Marco OLN, Millan LR, De Marco OLN, Rossi E, Arruda PCV (1999). Grupos de reflexão com quintanistas de medicina. O universo psicológico do futuro médico: vocação, vicissitudes e perspectivas.

[15] Yazdani M, Rezaei S, Pahlavanzadeh S (2010). The effectiveness of stress management training program on depression, anxiety and stress of the nursing students. Iran J Nurs Midwifery Res.

[16] Colares MFA, Andrade AS, Baldassin S (2012). Estudantes de medicina e atividade grupal reflexiva: uma prática possível. Atendimento psicológico aos estudantes de medicina: técnica e ética.

[17] Shapiro SL, Shapiro DE, Schwartz GE (2000). Stress management in medical education: a review of the literature. Academic medicine. J Assoc Am Med Coll.

[18] Baldassin S, Gomes A, Malbergier A, Gallassi AD, Andrade AS (2012). Atendimento psicológico aos estudantes de medicina: técnica e ética.

[19] Pereira MAD, Veiga WA, Ferreira ADC, Aguiar ASS, Guimarães CO, Fernandes LR (2006). Uma análise qualitativa dos fatores estressores e da qualidade de vida dos estudantes de medicina da Universidade Federal de Goiás. Rev Bras Educ Med.

[20] Pereira MAD, Barbosa MA (2013). Teaching strategies for coping with stress—the perceptions of medical students. BMC Med Educ.

[21] Goiás. Universidade Federal de Goiás—Resolução—CEPEC no 806. In: Goiânia. 2006. p 12.

[22] Sousa Filho PG (2009). Introdução aos métodos de relaxamento.

[23] Millan LR (2005). Vocação médica: um estudo de gênero.

[24] Bellodi PL, Baldassin S (2012). Tragédias, violência e trauma no curso médico: ecos nos serviços de apoio ao estudante de medicina. Atendimento psicológico aos estudantes de Medicina: técnica e ética.

[25] Mello Filho J (2006). Identidade médica.

[26] Guimarães KBS (2007). Saúde mental do médico e do estudante de medicina.

[27] Freud A (2006). O ego e os mecanismos de defesa.

[28] Lipp ME (2000). Manual do inventário de sintomas de stress para adultos de lipp (ISSL).

[29] Bardin L (2007). Análise de conteúdo.

[30] Yusoff MSB (2014). Interventions on medical students’ psychological health: a meta-analysis. J Taibah Univ Med Sci.

[31] Silva LCG, Rodrigues MMP (2004). Eventos estressantes na relação com o paciente e estratégias de enfrentamento: estudo com acadêmicos de medicina. J Bras Psiquiatr..

[32] Lee J, Graham AV (2001). Students’ perception of medical school stress and their evaluation of a wellness elective. Med Educ.

[33] de Almeida SC, Araújo RB (2005). Avaliação da efetividade do relaxamento na variação dos sintomas da ansiedade e da fissura em pacientes em tratamento de alcoolismo. Boletim da Saúde.

[34] Colares MFA, Andrade AS (2009). Atividades grupais reflexivas com estudantes de medicina. Rev Bras Educ Med..

